# On the Original Use of Musk

**Published:** 1810-09

**Authors:** 


### *To the Editors of the Medical and Physical Journal.*

GENTLEMEN,

THROUGH the medium of your very amusing and instructive Journal, I could wish to be informed, at what period of time Musk was first used, both as a medicine and as a perfume; also, in what country its use first originated. None of our more modern medical records, I believe, acquaint us with this. The Romans do not seem to have had a knowledge of the article, though they were familiar with other products of China. Did the Greeks employ it? Its name would appear to be of Greek derivation. I do not recollect any writer on the history of Musk, except it be Schroek, whose *Historia Moschi*, Vienna, 1682, I never could obtain a sight of.

I am, &c.

MEDICUS.

*August* 13, 1810.

